# ISTRF: Identification of sucrose transporter using random forest

**DOI:** 10.3389/fgene.2022.1012828

**Published:** 2022-09-12

**Authors:** Dong Chen, Sai Li, Yu Chen

**Affiliations:** ^1^ College of Electrical and Information Engineering, Qu Zhou University, Quzhou, China; ^2^ College of Information and Computer Engineering, Northeast Forestry University, Harbin, China

**Keywords:** machine learning, biological sequence analysis, protein identification, sucrose transporter, random forest

## Abstract

Sucrose transporter (SUT) is a type of transmembrane protein that exists widely in plants and plays a significant role in the transportation of sucrose and the specific signal sensing process of sucrose. Therefore, identifying sucrose transporter is significant to the study of seed development and plant flowering and growth. In this study, a random forest-based model named ISTRF was proposed to identify sucrose transporter. First, a database containing 382 SUT proteins and 911 non-SUT proteins was constructed based on the UniProt and PFAM databases. Second, k-separated-bigrams-PSSM was exploited to represent protein sequence. Third, to overcome the influence of imbalance of samples on identification performance, the Borderline-SMOTE algorithm was used to overcome the shortcoming of imbalance training data. Finally, the random forest algorithm was used to train the identification model. It was proved by 10-fold cross-validation results that k-separated-bigrams-PSSM was the most distinguishable feature for identifying sucrose transporters. The Borderline-SMOTE algorithm can improve the performance of the identification model. Furthermore, random forest was superior to other classifiers on almost all indicators. Compared with other identification models, ISTRF has the best general performance and makes great improvements in identifying sucrose transporter proteins.

## 1 Introduction

Sucrose is a kind of disaccharide, which is formed by the condensation of fructose and glucose molecules through dehydration and is widely found in various tissues of plants. In the process of plant photosynthesis, carbon transport is mainly in the form of sucrose (Kühn et al., 1999). Therefore, the distribution of sucrose directly affects the growth and yield of plants (Aluko et al., 2021; Mangukia et al., 2021). In terms of physical properties, sucrose is a non-reducing sugar, which can carry a large amount of carbon. In terms of chemical properties, its properties are very stable, and it is not easy to combine with other compounds during transportation, so it has a certain protective effect on carbon. In terms of biological properties, due to the carbon in sucrose having a higher osmotic potential, the transport speed of sucrose is faster in a sieve tube. Sucrose transporters affect the transport of sucrose, which is mainly distributed in parenchyma cells, companion cells, and vacuolar membranes. They are the mediators of sucrose transport from source leaves to the phloem. In addition, sucrose transporters also exist in sink organs, such as stems, seeds, and fruits. Sucrose transporters can promote sucrose transport under Pi starvation, salinity, and drought stress (Al-Sheikh Ahmed et al., 2018). At present, many experts have carried out a lot of research studies on sucrose transporters and found sucrose transporters in a variety of plant species, such as rice (Aoki et al., 2003), maize (Tran et al., 2017), grapevine, and tobacco (Wang et al., 2019). Endler et al. (2006) discovered a new sucrose transporter on the vacuolar membrane. They used liquid chromatography–tandem mass spectrometry to analyze tonoplast proteins and identified 101 proteins, including sucrose transporters. By studying the sucrose transporter gene RUSUT2 in blackberry, Yan et al. (2021) found that the sucrose content of mature leaves of the transgenic tobacco is enhanced by the overexpression of RUSUT2. At the same time, they found that Rusut2 has transport activity and may participate in sucrose transport during the growth and development of blackberry plants.

With the development of bioinformatics, more and more scholars used machine learning methods to identify sugar transporters. Mishra et al. (2014) developed a new model that incorporated the PSSM profile, amino acid composition, and biochemical composition of transporter proteins. The SVM algorithm was used as a classifier to classify transporters. Based on Mishra’s experiments, Alballa et al. (2020) used a series of features including position information, evolutionary information, and amino acid composition to improve the accuracy and MCC of transporter classification. Ho et al. (2019) used word embedding technology to extract effective features from protein sequences and then adopted traditional machine learning methods to classify a variety of transporters (including sugar transporters). It has been proved that machine learning can effectively solve some problems of protein classification. All of the above studies focused on sugar transporters, while Shah et al. proposed to use natural language processing technology BERT to carry out feature extraction of glucose transporters in sugar transporters and classify three glucose transporters through an SVM classifier (Shah et al., 2021). Using machine learning methods to identify special proteins has become a trend, and a machine learning frame has been employed to identify sugar transporters. All these previous works guide us to build a frame for identifying sucrose transporters. In this study, we constructed an identification model named ISTRF to identify sucrose transporters. First, a dataset is built. Second, protein sequences are encoded with k-separated-bigrams-PSSM. Third, the Borderline-SMOTE algorithm is used to augment the positive samples. Finally, the identification model is trained by the random forest algorithm.

## 2 Materials and methods

### 2.1 Frame chart of ISTRF

**FIGURE 1:**
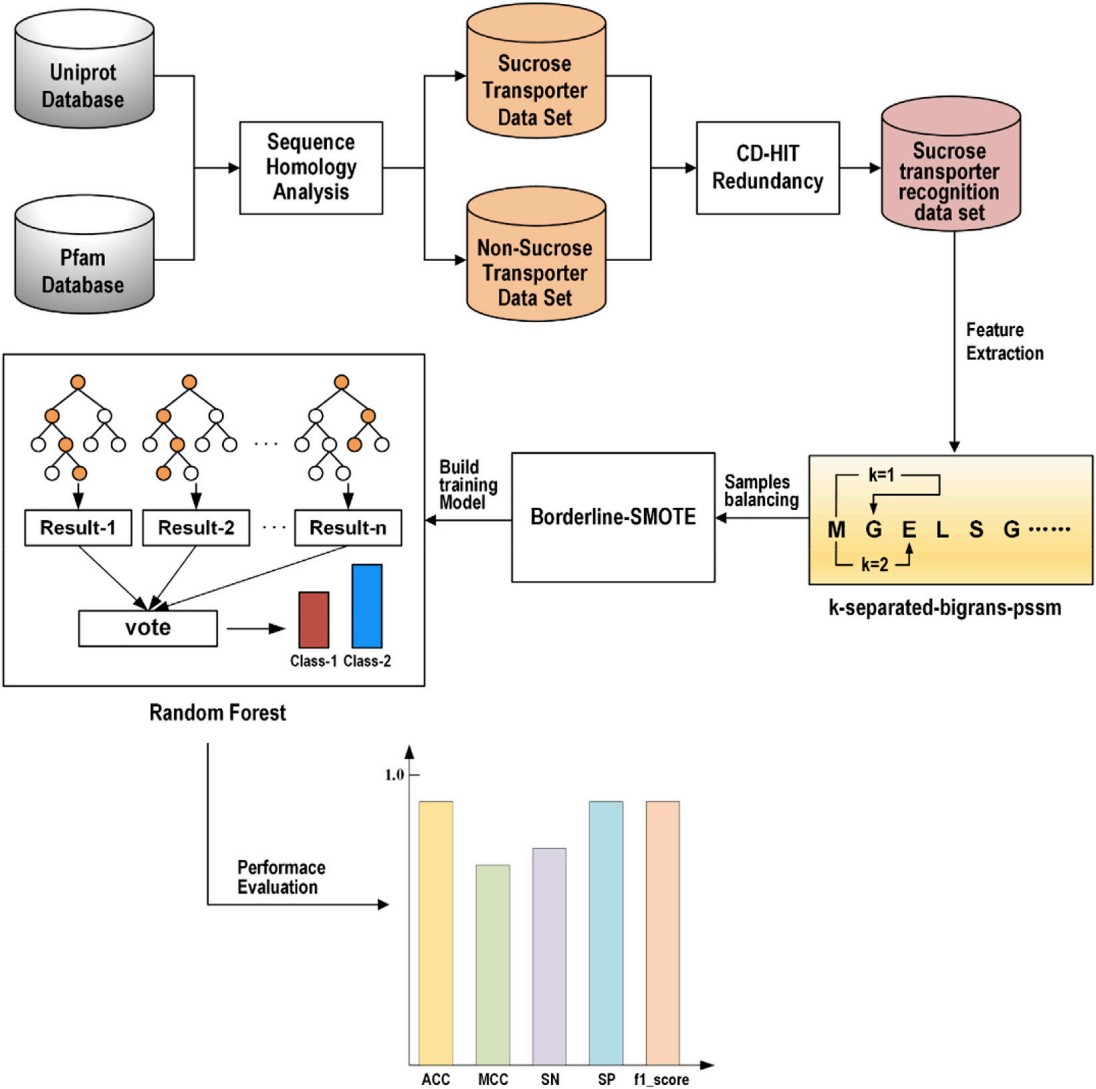
Frame chart of ISTRF.

In the study, we proposed a novel identification model called ISTRF, the frame chart of which is shown in Figure 1. First of all, the sucrose and non-sucrose transporter datasets are obtained using sequence homology analysis technology based on the Uniprot and Pfam databases, and then the CD-HIT program was used to remove redundancy and delete the protein sequences with more than 60% similarity. The sucrose transporter samples are construed for the training identification model. Second, we extracted the k-separated-bigrams-PSSM feature to represent samples. Third, we augment the positive samples to balance the training samples by using the Borderline-SMOTE technology. Finally, we built a random forest-based classifier that takes the balancing feature vectors as input. In the following sections, the dataset, feature extraction, sample balancing, and classifiers will be, respectively, introduced in detail.

### 2.2 Dataset

In this study, a self-built dataset is constructed and used. To obtain a reliable experimental result, it is necessary to use a high-quality benchmark data set, and then the initial data must be processed strictly and standardly. UniProt (Consortium, 2019) database is an authoritative protein database, in which we searched by the keyword “sucrose transporter” to obtain the initial positive sample data set. From the protein family database PFAM (Mistry et al., 2021), families containing positive samples were deleted, and the protein sequence with the longest length was extracted from every remaining family as a negative sample to construct the initial negative sample data set. Next, we processed the initial data set. The first step was to delete the protein sequences containing illegal characters; the second step was to delete the protein sequences with a length less than 50; in the third step, the CD-HIT (Fu et al., 2012) program was used to remove redundancy and delete the protein sequences with more than 60% similarity. We eventually obtained 382 SUTs and 9,109 non-SUTs. This data set is extremely unbalanced, so we divided the negative sample data set into ten equally and took one as the experimental data, which is 911 non-SUTs. We divided the data set into an 80% training dataset and a 20% testing dataset and constructed the dataset as shown in Table 1.

**TABLE 1:** Self-built dataset.

Dataset	SUT	Non-SUT
Training dataset	306	729
Testing dataset	76	182

### 2.3 Feature extraction

In the process of protein identification, feature extraction is a crucial step (Yang and Jiao, 2021). To improve the identification performance of the model, we tried to extract features with high identification and good specificity. In this study, we considered this problem from two perspectives, namely, physicochemical properties and evolutionary information. We tried three features and their various combinations. Finally, the k-separated-bigrams-PSSM which has the best performance according to the experimental result is selected as the feature representation method in our model.

#### 2.3.1 188D

188D includes the frequency of 20 amino acids and eight physical and chemical properties (Cai et al., 2003).

The formula for calculating the frequency of 20 amino acids is as follows:

$$F_{i}=\frac{N_{i}}{L},\left(i=A,C,D,\ldots ,Y\right),$$

where N_i_ is the number of amino acid type i, and L is the length of a protein sequence.

The composition, transition, and distribution are used to describe eight physicochemical properties of proteins (Xiong et al., 2018; Zou et al., 2019; Masoudi-Sobhanzadeh et al., 2021). Taking the hydrophobicity attribute as an example, “RKEDQN” is polar, “GASTPHY” is neutral, and “CVLIMFW” is hydrophobic. The frequency of each group can be expressed as follows:

$$C_{i}=\frac{N_{i}}{L},i\in \left\{polar,neutral,hydrophobic\right\}.$$

The transition from polar group to neutral group is the frequency of polar residue following neutral residue or neutral residue following polar residue. The transition between the neutral group and hydrophobic group and the transition between the hydrophobic group and polar group have similar definitions. It can be expressed by the following formula:

$$\begin{array}{l} T\left(i_{1},i_{2}\right)=\frac{N\left(i_{1},i_{2}\right)+N\left(i_{2},i_{1}\right)}{L-1},\left(i_{1},i_{2}\right)\in \\ \left\{\left(polar,neutral\right),\left(neutral,hydrophobic\right),\left(hydrophobic,polar\right)\right\}. \end{array}$$

The distribution consists of five values, which are the first, 25, 50, 75, and 100% positions of each group of amino acid in the sequence.

#### 2.3.2 PSSM composition

PSSM composition is a feature that describes the evolutionary information of protein sequences, and it is also used to identify a variety of proteins (Wang et al., 2018; Ali et al., 2020; Qian et al., 2021). First, we run the PSI-BLAST tool (Ding et al., 2014) against the Uniref50 database with the e-value set to 0.001. We can obtain the original PSSM profile. Then, we summed the same amino acid rows together and divided the results by the number of amino acids in the protein sequence. Finally, a 400-dimensional PSSM composition was obtained.

#### 2.3.3 The k-separated-bigrams-PSSM

The k-separated-bigrams-PSSM is generated from the original PSSM profile by column transformation. It represents the transition probabilities from one amino acid to another amino acid in a protein sequence (Wang et al., 2020), and the interval of the two amino acids is K. N represents the PSSM matrix, and L is the number of amino acids in the protein sequence and also the number of rows in the PSSM matrix. The transition from the m-th amino acid to the n-th amino acid can be expressed by the following formulas:

$$T_{m,n}\left(k\right)=\overset{L-k}{\underset{i=1}{\sum }}N_{i,m}N_{i+k,n},$$

where 1 ≤ m ≤ 20, 1 ≤ n ≤ 20, and 1 ≤ k ≤ K.

$$T\left(k\right)=\left[T_{1,1}\left(k\right),T_{1,2}\left(k\right),\ldots ,T_{1,20}\left(k\right),T_{2,1}\left(k\right),\ldots ,T_{2,20}\left(k\right),\ldots ,T_{20,1}\left(k\right),\ldots ,T_{20,20}\left(k\right)\right].$$

For each k, T(k) is a 400-dimensional feature that represents 400 amino acid transitions. The k ranges from 1 to 11. When k is set to 1, it represents the transition probabilities between neighboring amino acids; when k is set to 2, it represents the transition probabilities between amino acids with one amino acid between them. We can obtain k-separated-bigrams-PSSM (k = 1) and a PSSM-related transformation matrix through POSSUM (Wang et al., 2017). The website is open, and users can easily obtain the required features.

### 2.4 Sample balancing

The training dataset constructed in Section 2.2 is an imbalance dataset, on which the classifier trained is biased to identify the unseen sample as the majority class (Shabbir et al., 2021). Therefore, we use the Borderline-SMOTE algorithm to balance the feature set. The SMOTE (Chawla et al., 2002) algorithm is an oversampling technique for synthesizing minority classes. It uses the KNN algorithm to calculate the k nearest neighbors of each minority class sample, randomly selects N samples, and performs random linear interpolation on the k nearest neighbors to construct new minority class samples. However, it does not consider the position of the adjacent majority class samples, which usually leads to the phenomenon of sample overlap and affects the classification effect (Chen et al., 2021). Borderline-SMOTE (Han et al., 2005) is an improved oversampling algorithm based on SMOTE. Because the boundary samples are more likely to be misclassified than those far from the boundary, the algorithm only oversamples the boundary samples of the minority class. In the Borderline-SMOTE algorithm, we used the KNN algorithm with k = 5 to balance the feature set of sucrose transporters, so that the 306 SUTs and 729 non-SUTs in the training set were expanded to 729 SUTs and 729 non-SUTs.

### 2.5 Classifier

In this study, we tried a lot of classification algorithms such as SVM, naive Bayes, SGD, and random forest (Ao et al., 2022). Eventually, we selected the random forest as our classifier based on the experimental results shown in Section 3.3. These machine learning algorithms can be implemented by the WEKA (Holmes et al., 1994; Garner, 1995) software. WEKA is an open data mining platform that can perform data processing such as classification, regression, and clustering. It contains a variety of machine learning algorithms and is simple to operate.

SVM is a supervised learning algorithm and is implemented by the SMO (sequential minimal optimization) algorithm in WEKA (Vapnik, 2006). The classical SVM algorithm has been applied to many problems of bioinformatics, especially in binary classification (Manavalan et al., 2018; Zhang et al., 2019; Ao et al., 2021; Zeng et al., 2021). The main idea is to find an optimal segmentation hyperplane and measure the maximum geometric distance between the nearest sample and the hyperplane so as to divide the data set correctly. The SMO algorithm is an improved support vector machine algorithm that aims to improve the efficiency of the support vector machine. It breaks the large quadratic programming (QP) problem into many smaller QP problems and avoids the problem that the time-consuming numerical QP optimization is used in the inner loop (Platt, 1998).

Naive Bayes is a very classical and simple classification algorithm (Cao et al., 2003). The idea of the algorithm is also very simple. For a given sample to be classified, the probability that it belongs to the positive sample and the negative sample is solved firstly. And then the sample will be classified into the category with the higher probability. It assumes that each input variable is independent. Although real life cannot meet this assumption, it is still valid for most complex problems.

Stochastic gradient descent (SGD) is often used to learn linear classifiers under convex loss functions such as logistic regression and support vector machines (Bottou, 2010). The SGD algorithm is proposed to solve the problem that batch gradient descent needs to use all the samples for each parameter update, and the speed is slow when the number of samples is large. The characteristic of the SGD algorithm is that in each iteration, a group of samples is randomly chosen for training. After N iterations, it finds out the coefficient which leads to the minimum error of these models.

Random forest is based on the idea of ensemble learning, and it integrates multiple decision trees to obtain classification results (Breiman, 2001). First of all, select k samples repeatedly and randomly from the original training sample set N to generate a new training sample set. Then, n decision trees are generated using the training sample set as input. These decision trees form a random forest. Each decision tree is a classifier. As many decision trees as there are, there are as many classification results. Finally, the random forest integrates the classification results of n decision trees and identifies the class with most votes as the classification result of the sample. Because of this randomness, the random forest has a good anti-noise capability and is very suitable for processing high-dimensional data and avoiding overfitting. In many studies, random forest has shown a good classification effect (Lv et al., 2019; Ru et al., 2019; Ao et al., 2020; Lv et al., 2020; Petry et al., 2020; Zhang et al., 2021a).

### 2.6 Measurement

We used five indicators to evaluate the performance of our identification model: sensitivity (SN), specificity (SP), accuracy (ACC), Marshall correlation coefficient (MCC), and F-measure (Basith et al., 2020; Zhang et al., 2021b; Lee et al., 2021). These evaluation indicators were the results of the confusion matrix calculation obtained from the experiment, and the calculation formula is as follows:

$$SN=\frac{TP}{TP+FN}$$

$$SP=\frac{TN}{TN+FP}$$

$$ACC=\frac{TP+TN}{TP+TN+FP+FN}$$

$$MCC=\frac{\left(TP\times TN\right)-\left(FP\times FN\right)}{\sqrt{\left(TP+FP\right)\times \left(TP+FN\right)\times \left(TN+FP\right)\times \left(TN+FN\right)}}$$

$$FR=\frac{TP}{TP+FP}$$

$$F-Measure=\frac{2\times SN\times \mathrm{PR}}{SN+\mathrm{PR}}$$

where TP represents the number of correctly predicted sucrose transporters, TN represents the number of correctly predicted non-sucrose transporters, FP represents the number of incorrectly predicted sucrose transporters as non-sucrose transporters, and FN represents the number of incorrectly predicted non-sucrose transporters as sucrose transporters.

## 3 Results and discussion

### 3.1 Performance of different features

As shown in the frame chart of ISTRF in Section 2.1, our model extracted the k-separated-bigrams-PSSM feature to encode samples. To prove the effectiveness of our feature extraction method, we conducted experiments to compare the performance of different feature extraction algorithms. Specifically, we selected 188D, PSSM composition, k-separated-bigrams-PSSM, and their combinations. 188D feature reflected the frequency of 20 amino acids and eight physical and chemical properties, while PSSM composition and k-separated-bigrams-PSSM reflected the evolutionary information of protein sequences. We used the random forest as a classifier and did not apply Borderline-SMOTE to the extracted feature, and the experimental results of different features on 10-fold cross-validation are shown in Table 2. Bold values in the table indicate the best results. According to the number of indicators with the highest value, the number of k-separated-bigrams-PSSM is 4, the number of combinational features of 188D and k-separated-bigrams-PSSM is 4, and the number of other features and combinational features is lower or equal to 1. The k-separated-bigrams-PSSM has fewer feature numbers than the combination of 188D and k-separated-bigrams-PSSM; therefore, the former has the best performance according to the number of indicators with the highest value. According to the indicator of ACC and MCC, k-separated-bigrams-PSSM still has the highest value, and it verified that k-separated-bigrams-PSSM has the best general performance. Considering the indicator of SN, our used k-separated-bigrams-PSSM also has the maximum value. It verifies that our feature extraction method has better performance than other methods in predicting sucrose transporter protein from positive examples. Considering the indicator of SP, our feature extraction method is slightly lower than the combinational feature of PSSM composition and k-separated-bigrams- PSSM and is equal to or higher than other methods. However, the indicators of SN, MCC, and ACC of our feature extraction method are obviously larger than the combinational feature of PSSM composition and k-separated-bigrams-PSSM, which verify that the combinational feature of PSSM composition and k-separated-bigrams-PSSM trends to identify a protein as a non-sucrose transporter protein. Based on the fact that training data are an unbalanced data set in which negative samples are larger than positive ones, our feature extraction method is less affected by unbalanced data. After balancing the training data, the SN of our feature method is larger than the combinational feature of PSSM composition and k-separated-bigrams-PSSM, and the detailed experimental results are shown in Section 3.2. Therefore, from the overall perspective, our method obviously performs better than all other methods.

**TABLE 2:** Result of various feature extraction methods using random forest without Borderline-SMOTE on 10-fold cross-validation.

Feature	SN	SP	ACC	MCC	F-measure
188D	0.895	0.970	0.948	0.874	0.910
PSSM composition	0.876	0.967	0.940	0.855	0.896
k-separated-bigrams-PSSM	**0.925**	0.973	**0.958**	**0.900**	**0.929**
188D + PSSM composition	0.895	0.973	0.950	0.878	0.913
188D + k-separated-bigrams-PSSM	**0.925**	0.973	**0.958**	**0.900**	**0.929**
PSSM composition + k-separated-bigrams-PSSM	0.908	**0.978**	0.957	0.897	0.927
188D + PSSM composition + k-separated-bigrams-PSSM	0.918	0.973	0.957	0.895	0.926

Bold values in the table indicate the best results.

To further illustrate that our feature extraction method also has better performance using other classifiers, we also conducted experiments on different features using an SGD classifier. Table 3 shows the experimental results. As we can see from Table 3, our feature extraction method has better performance than other methods according to the number of indicators with the highest value or ACC indicator or MCC indicator. All in all, our feature extraction method performs better than other feature extraction methods.

**TABLE 3:** Result of various feature extraction methods using SGD without Borderline-SMOTE on 10-fold cross-validation.

Feature	SN	SP	ACC	MCC	F-measure
188D	0.866	0.951	0.926	0.821	0.873
PSSM composition	0.873	0.956	0.931	0.834	0.883
k-separated-bigrams-PSSM	**0.964**	0.952	**0.956**	**0.897**	**0.928**
188D + PSSM composition	0.902	0.959	0.942	0.861	0.902
188D + k-separated-bigrams-PSSM	0.912	0.952	0.940	0.857	0.900
PSSM composition + k-separated-bigrams-PSSM	0.905	**0.967**	0.949	0.877	0.913
188D + PSSM composition + k-separated-bigrams-PSSM	0.912	0.960	0.946	0.870	0.909

Bold values in the table indicate the best results.

### 3.2 Experiments on sample balancing

As shown in the frame chart of ISTRF in Section 2.1, the sucrose transporter database built in this study has more negative samples than positive ones, and it is an imbalanced dataset that influences the classification performance of the machine learning algorithm. We adopted Borderline-SMOTE to augment the positive samples, and finally the number of positive samples is equal to negative samples. To verify that Borderline-SMOTE is effective for our model, we, respectively, conducted experiments using random forest and SGD on the basis of different features with Borderline-SMOTE. Experimental results are shown in Tables 4 and 5. The first number is the experimental result using Borderline-SMOTE, the second number is the percentage of increase or decrease relative to one without Borderline-SMOTE, and the plus sign denotes an increase, while the minus sign denotes a decrease. By comparing Table 4 with Table 2, we can see that the performance of features using Borderline-SMOTE is better than features not using Borderline-SMOTE in all indicators except indicator SP. The same conclusion is also obtained by comparing Table 5 with Table 3. In general, the features of Borderline-SMOTE can improve classification performance.

**TABLE 4:** Result of various features using random forest with Borderline-SMOTE on 10-fold cross-validation.

Feature	SN	SP	ACC	MCC	F-measure
188D	0.989 + 9.4	0.937–3.3	0.963 + 1.5	0.927 + 5.3	0.964 + 5.4
PSSM composition	0.982 + 10.6	0.952–1.5	0.967 + 2.7	0.935 + 8	0.968 + 7.2
k-separated-bigrams-PSSM	0.986 + 6.1	0.970–0.3	0.978 + 2	0.956 + 5.6	0.978 + 4.9
188D + PSSM composition	0.982 + 8.7	0.952–2.1	0.967 + 1.7	0.935 + 5.7	0.968 + 5.5
188D + k-separated-bigrams-PSSM	0.984 + 5.9	0.945–2.8	0.964 + 0.6	0.929 + 2.9	0.965 + 3.6
PSSM composition + k-separated-bigrams-PSSM	0.984 + 7.6	0.957–2.1	0.971 + 1.4	0.941 + 4.4	0.971 + 4.4
188D + PSSM composition + k-separated-bigrams-PSSM	0.985 + 6.7	0.949–2.4	0.967 + 1	0.935 + 4	0.968 + 4.2

**TABLE 5:** Result of various features using SGD with Borderline-SMOTE on 10-fold cross-validation.

Feature	SN	SP	ACC	MCC	F-measure
188D	0.966 + 10	0.909–4.2	0.938 + 1.2	0.877 + 5.6	0.939 + 6.6
PSSM composition	0.975 + 10.2	0.938–1.8	0.957 + 2.6	0.914 + 8	0.958 + 7.5
k-separated-bigrams-PSSM	0.997 + 3.3	0.942–1	0.970 + 1.4	0.941 + 4.4	0.971 + 4.3
188D + PSSM composition	0.985 + 8.3	0.931–2.8	0.958 + 1.6	0.918 + 5.7	0.959 + 5.7
188D + k-separated-bigrams-PSSM	0.985 + 7.3	0.931–2.1	0.958 + 1.8	0.918 + 6.1	0.959 + 5.9
PSSM composition + k-separated-bigrams-PSSM	0.984 + 7.9	0.951–1.6	0.967 + 1.8	0.945 + 6.8	0.968 + 5.5
188D + PSSM composition + k-separated-bigrams-PSSM	0.988 + 7.6	0.940–2	0.964 + 1.8	0.928 + 5.8	0.965 + 5.6

**FIGURE 2:**
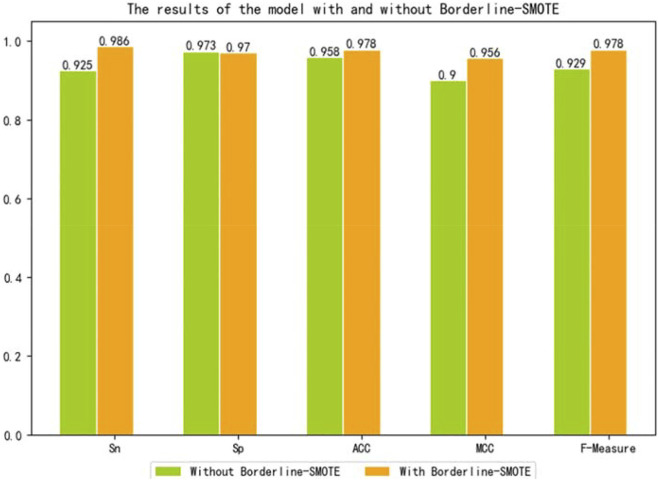
Results of the model with or without Borderline-SMOTE on 10-fold cross-validation.

**FIGURE 3:**
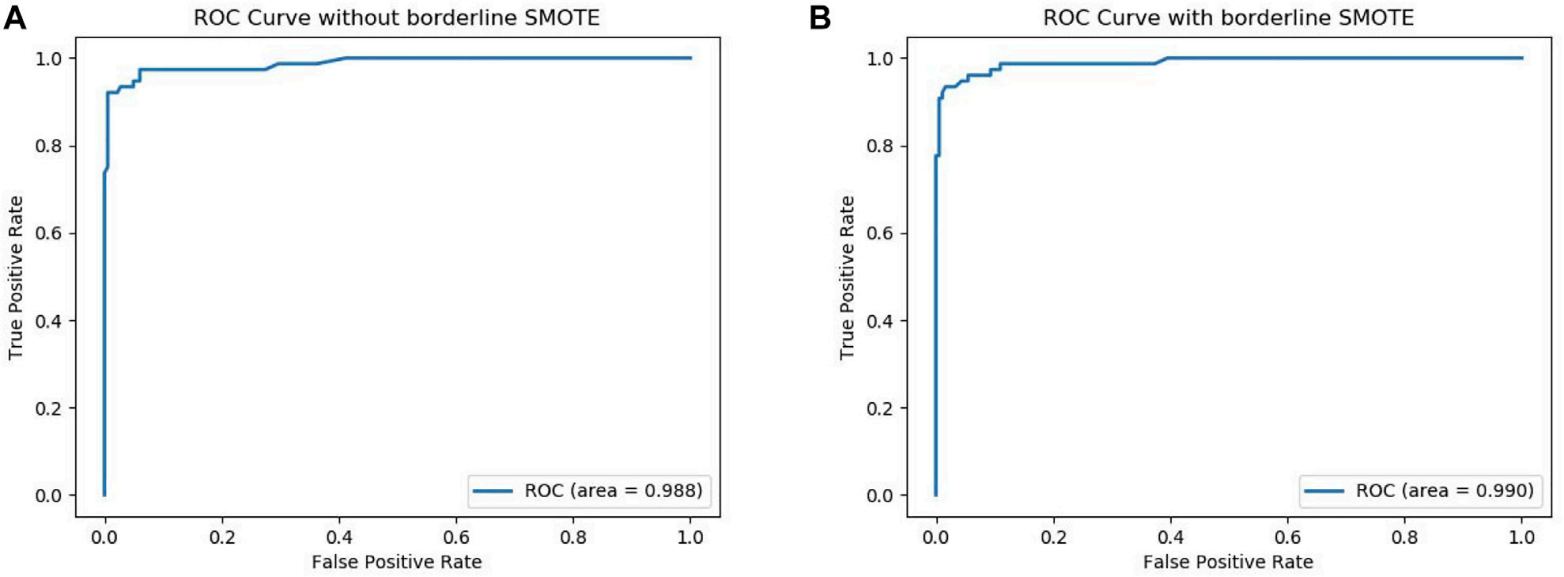
ROC curve with or without Borderline-SMOTE. **(A)** ROC curve without Borderline-SMOTE. **(B)** ROC curve with Borderline-SMOTE.

To further verify that our model can use Borderline-SMOTE to improve the classification performance, that is, Borderline-SMOTE is effective in our model. We compared the performance of our model with Borderline-SMOTE and without Borderline-SMOTE on 10-fold cross-validation, and the experimental result is shown in Figure 2. Except for a slight decrease in SP, all other indicators improved by 2.0–6.1% in Figure 2, especially the indicator SN, which improved to its maximum. The decrease of SP verified that Borderline-SMOTE avoids our model being biased to classifying samples into negative samples. The increase of SN verified that Borderline-SMOTE improves our model’s identification ability of positive samples. The improvement of indicators of ACC, MCC, and F-measure verified that Borderline-SMOTE improved our model performance from a general perspective. Furthermore, the ROC curves of our model are plotted in Figure 3, and it can be seen that ISTRF with Borderline-SMOTE is superior to ISTRF without Borderline-SMOTE in the prediction of sucrose transporter protein.

**FIGURE 4:**
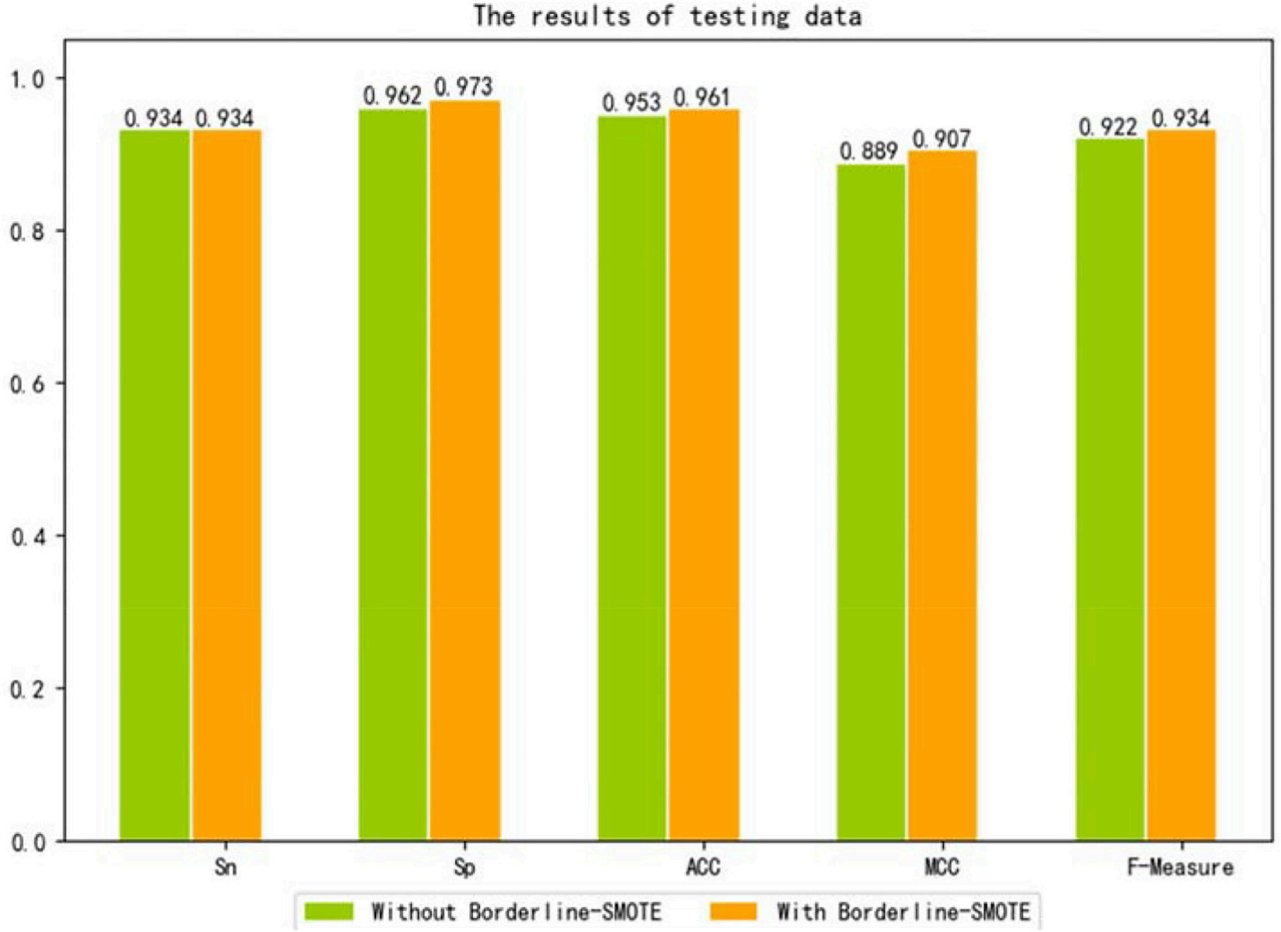
Results of the model with or without Borderline-SMOTE on the test dataset.

To further evaluate the performance of Borderline-SMOTE in an unseen data set, we conducted experiments on the unseen data set. We used the testing set containing 76 sucrose transporters and 182 non-sucrose transporters to verify the model, and the experimental result is shown in Figure 4. By comparing the two models without and with Borderline-SMOTE, it was found that the latter performs better, which proves once again that Borderline-SMOTE improves the performance of our model.

### 3.3 Performance of various classifiers

As shown in the frame chart of ISTRF, we adopt random forest as a classifier to train the identification model. To verify that random forest has a better performance than other classifiers, we compared random forest with SVM, NB, and SGD. Table 6 showed the experimental result of 10-fold cross-validation using the k-separated-bigrams-PSSM feature without the Borderline-SMOTE as input. Table 7 showed the experimental result of 10-fold cross-validation using the k-separated-bigrams-PSSM feature with the Borderline-SMOTE as input.

**TABLE 6:** Result of various classifiers using k-separated-bigrams-PSSM feature without Borderline-SMOTE on 10-fold cross-validation.

Classifier	SN	SP	ACC	MCC	F-measure
SVM	0.948	0.944	0.945	0.872	0.911
NB	**0.984**	0.782	0.842	0.703	0.786
SGD	0.964	0.952	0.956	0.897	0.928
RF	0.925	**0.973**	**0.958**	**0.900**	**0.929**

Bold values in the table indicate the best results.

**TABLE 7:** Result of various classifiers using k-separated-bigrams-PSSM feature with Borderline-SMOTE on 10-fold cross-validation.

Classifier	SN	SP	ACC	MCC	F-measure
SVM	**0.997**	0.877	0.937	0.880	0.940
NB	0.989	0.774	0.881	0.781	0.893
SGD	0.997	0.942	0.970	0.941	0.971
RF	0.986	**0.970**	**0.978**	**0.956**	**0.978**

Bold values in the table indicate the best results.

In Table 6, although the random forest classifier is slightly lower than BN on the SN indicator, it is obviously superior to the other four indicators. According to the number of indicators with the highest value, random forest obtained the four highest values and performs better than the compared classifiers. It is seen in Table 7 that random forest also performs better than the compared classifiers. All in all, random forest is effective in identifying sucrose transporter proteins.

### 3.4 Comparison with existing methods

To further evaluate the performance of ISTRF, our model is compared with the existing prediction method BioSeq-Analysis (Liu et al., 2019). The online address for this method is http://bioinformatics.hitsz.edu.cn/BioSeq-Analysis/PROTEIN/Kmer/. The SVM and random forest algorithms are used in the BioSeq-Analysis prediction method. We compared them separately. The prediction results are shown in Table 8. It can be seen from Table 8 that our identification model outperforms the compared models on the indicators of ACC, MCC, and SN. It verified that our identification model performs better in general.

**TABLE 8:** Experimental result of using different methods.

Model	ACC	MCC	SN	SP
ISTRF	**0.961**	**0.907**	**0.934**	0.973
BioSeq-SVM	0.9457	0.8694	0.9079	0.9615
BioSeq-RF	0.938	0.8505	0.8026	**0.9945**

Bold values in the table indicate the best results.

## 4 Conclusion

A large number of experiments have proved that sucrose transporters play an important role in plant growth and crop yield. Therefore, the identification of sucrose transporters has become particularly important. With the rapid development of high-throughput sequencing technology, protein sequences can be easily obtained. In contrast, traditional biochemical technology needs a lot of human, material, and financial resources, and the identification of proteins through bioinformatics methods has become a popular trend. In this study, we introduced k-separated-bigrams-PSSM as the input feature, random forest as the classifier, and the Borderline-SMOTE algorithm to balance the training set. We achieved 0.978 accuracy, 0.986 SN, 0.970 SP, 0.956 MCC, and 0.978 F-measure on the training set. In order to verify the effectiveness of the model, the testing set was used for experiments, and the accuracy was 0.961. In the future, we will continue to find breakthroughs, optimize the experimental model, and strive to obtain better results.

## Data Availability

The original contributions presented in the study are included in the article/Supplementary Material; further inquiries can be directed to the corresponding author.
